# Supplementary material for: The structural context of posttranslational modifications at a proteome-wide scale
Source: PLoS Biol. 2022 May 16;20(5):e3001636. doi: 10.1371/journal.pbio.3001636 (PMC9135334; doi:10.1371/journal.pbio.3001636)
Supplement: Attachment — Submitted filename: Point_by_point_response.docx [file pbio.3001636.s008.docx]

April 13, 2022

**Response to Reviewers**

We thank the editors and reviewers for their thorough evaluation of our work and their many insightful comments. We were delighted by the overall positive feedback. Please find our detailed point-by-point responses *in line* below.

From the editor:

Dear Matthias,

Thank you for your patience while we peer-reviewed your manuscript "The structural context of PTMs at a proteome wide scale" as a Methods and Resources study at PLOS Biology. Your manuscript has been evaluated by the PLOS Biology editors, an Academic Editor with relevant expertise, and by several independent reviewers. I have taken over the handling of tis submission during Richard's absence from the office this week, to prevent any unnecessary loss of time.

As you will see from the reviews at the end of this email, all of the reviewers are very supportive of the work. Reviewers 2 and 3 have identified themselves as Jon Agirre and Julian Langer, respectively. Reviewer 1 has raised more critical concerns despite the overall support, and upon discussion with our Academic Editor we consider the request to look at deposited ensembles in the PEDB to be a good one, but if there are not enough submissions for the analyses, then the concerns could be addressed by textual changes by clarifying the limitations/parameters of the 3D hotspot model.

We investigated ensemble structures deposited in the PEDB and indeed realized that there are only 242 ensembles available to date (Lazar et al, 2021), which means that any global statistical analysis would be substantially limited. Based on the comments of reviewer 1, we assume that the use of ‘static’ structures is their main concern. We agree that it would be problematic if one considered the AlphaFold structures only as static. However, we do not think this is fully the case as we specifically use the predicted aligned error (PAE) between any two amino acids for all distance calculations, including pPSE values and 3D proximity. The PAE essentially provides a measure of uncertainty between the relative position of amino acids, which is generally low within folded domains and large between amino acids in IDRs or across domains that are linked by IDRs, as these can move relative to each other. We therefore expect our PAE-based distance calculations to account for non-‘static’ protein regions somewhat similar to considering PEDB ensembles. We edited the main text in both the result and discussion section to provide more insights into the analysis rational and to clearly state the limitations of the current approach. Please also see our direct response to reviewer 1 for further details and the specific text segments that we added.

In light of the reviews, we are pleased to offer you the opportunity to address all of the concerns raised by the reviewers in a revised version that we anticipate should not take you very long. We will then assess your revised manuscript and your response to the reviewers' comments with our Academic Editor and may consult (as subset of) the reviewers again. We expect to receive your revised manuscript within 1 month, but please let us know if you would need longer.

In addition, please ensure that your revision also addresses the following reporting and/or formatting concerns (this will be needed for eventual acceptance, so doing this now will speed up the process):

- it is PLOS policy that, as a condition of publication, you make available all data used to draw the conclusions arrived at in your manuscript (<http://journals.plos.org/plosbiology/s/data-availability>). The summary numerical values underlying the graphs all of the main and supplementary figures should be provided, either in a source data file within the manuscript's supplementary information or, preferably, in a public repository. All figure legends must then include information on where the underlying source data for the figure can be found (if this is a repository, the name of the specific file needs to be indicated, rather than e.g. a folder or directory). Please note that this does not refer to the raw data analysed, which we also encourage to provide, but the summary numerical values that are directly related to the figures shown.

We created a GitHub repository (https://github.com/MannLabs/structuremap_analysis) which contains all data and python Jupyter notebooks for performing all described analyses and for generating the figures in the paper. We added links to the source data for each Figure. However, we find the long URL links very unpleasant when reading the Figure captions. If possible, it would be great if short links could be created for each file to avoid the long URLs.

- would you be able to add an explanation for the grey column in fig. 2B in the figure legend? It strikes as a bit odd that the other columns are based on specific references, but then 677 papers are shown together. I realise that this explanation may be elsewhere in the manuscript, but a lot of readers focus on the figures and legends, so it should also be there.

We included an explanation of the grey color bar in the figure caption. The revised caption for Figure 1B now states the following:

“**(*B)*** *Overview of datasets that contribute ubiquitination sites to PhosphoSitePlus. Publications reporting > 1000 ubiquitination sites are listed separately and are colored based on their use of proteasome inhibitors. The additional 677 studies that contribute fewer ubiquitination sites were aggregated to a grey bar irrespective of their use of proteasome inhibitors.*”

- the references are not in PLOS Biology format; please see [https://journals.plos.org/plosbiology/s/submission-guidelines#loc-formatting-references](https://journals.plos.org/plosbiology/s/submission-guidelines#loc-formatting-references)

We updated the reference style according to the PLOS Biology guidelines.

- the author contributions, and conflict of interest statements will be taken from the information that you enter in our submission system for publication. Please delete these sections from the manuscript, but when you submit your revision, ensure the information in the submission system is complete and accurate

We have done this.

- likewise, in the financial disclosure information (which is also on our electronic submission system), please ensure that all sources of funding are identified with the relevant ID/number

Also done.

- Matthias, we have fixed the duplicate account issue, and so you should now be able to link your ORCiD to the submission while submitting the revised version without a problem (we cannot do this bit on your behalf) - do let me know if this is not solved, but I am told it is

Thank you very much.

Please email us ([plosbiology@plos.org](mailto:plosbiology@plos.org)) if you have any questions or concerns about the revision. At this stage, your manuscript remains formally under active consideration at our journal; please notify us by email if you do not intend to submit a revision so that we may end consideration of the manuscript at PLOS Biology.

**IMPORTANT - SUBMITTING YOUR REVISION**

Your revisions should address the specific points made by each reviewer. Please submit the following files along with your revised manuscript:

1. A 'Response to Reviewers' file - this should detail your responses to the editorial requests, present a point-by-point response to all of the reviewers' comments, and  indicate the changes made to the manuscript. 
*NOTE: In your point by point response to the reviewers, please provide the full context of each review. Do not selectively quote paragraphs or sentences to reply to. The entire set of reviewer comments should be present in full and each specific point should be responded to individually.
You should also cite any additional relevant literature that has been published since the original submission and mention any additional citations in your response. 

2. In addition to a clean copy of the manuscript, please also upload a 'track-changes' version of your manuscript that specifies the edits made. This should be uploaded as a "Related" file type. 

*Resubmission Checklist*
When you are ready to resubmit your revised manuscript, please refer to this resubmission checklist: <https://plos.io/Biology_Checklist>


To submit a revised version of your manuscript, please go to <https://www.editorialmanager.com/pbiology/> and log in as an Author. Click the link labelled 'Submissions Needing Revision' where you will find your submission record. 

Please make sure to read the following important policies and guidelines while preparing your revision:

*Published Peer Review*
Please note while forming your response, if your article is accepted, you may have the opportunity to make the peer review history publicly available. The record will include editor decision letters (with reviews) and your responses to reviewer comments. If eligible, we will contact you to opt in or out. Please see here for more details:
<https://blogs.plos.org/plos/2019/05/plos-journals-now-open-for-published-peer-review/>

*PLOS Data Policy*
Please note that as a condition of publication PLOS' data policy (<http://journals.plos.org/plosbiology/s/data-availability>) requires that you make available all data used to draw the conclusions arrived at in your manuscript. If you have not already done so, you must include any data used in your manuscript either in appropriate repositories, within the body of the manuscript, or as supporting information (N.B. this includes any numerical values that were used to generate graphs, histograms etc.). For an example see here: [http://www.plosbiology.org/article/info%3Adoi%2F10.1371%2Fjournal.pbio.1001908#s5](http://www.plosbiology.org/article/info%3Adoi%2F10.1371%2Fjournal.pbio.1001908#s5) 

*Protocols deposition*
To enhance the reproducibility of your results, we recommend that if applicable you deposit your laboratory protocols in [protocols.io](http://protocols.io), where a protocol can be assigned its own identifier (DOI) such that it can be cited independently in the future. Additionally, PLOS ONE offers an option for publishing peer-reviewed Lab Protocol articles, which describe protocols hosted on [protocols.io](http://protocols.io). Read more information on sharing protocols at <https://plos.org/protocols?utm_medium=editorial-email&utm_source=authorletters&utm_campaign=protocols>  


Thank you again for your submission to our journal. We hope that our editorial process has been constructive thus far, and we welcome your feedback at any time. Please don't hesitate to contact us if you have any questions or comments.

With best wishes,

Nonia


Nonia Pariente, PhD
Editor in Chief
PLOS Biology


on behalf of

Richard


Richard Hodge
Associate Editor
PLOS Biology
[rhodge@plos.org](mailto:rhodge@plos.org)

*****************************************************

REVIEWS:

Reviewer #1: 
The authors report results from their analysis of sites for PTMs that are based on the use of predicted protein structures. The latter were derived from AlphaFold. The question, as it comes through in my reading of the MS, is the extent to which predicted structures hold insights regarding the different PTM patterns. For this, the authors develop metrics for different types of PTMs, based largely on accessibility of the different sites for different PTMs, and evaluate their results based on extant knowledge bases. The results corroborate what has been established regarding phosphorylation. The results do, however, offer interesting and novel insights regarding what we think we know about ubiquitination and acetylation. 

Some of the findings, especially those pertaining to Ser / Thr phosphorylation are not really surprising or novel. This has a lot to do with the extents to which these modifications have been over-studied. It would help to have a crisp summary of the novel findings that emerge. For example, it is well established (sorry for using this trope) that phosphosites are enriched within IDRs. What more do we learn from the current analysis? The answer to this question was not entirely clear. One helpful insight was the comparative analysis of phosphosites gleaned from the use of structure predictions vs. the analysis of Sugiyama et al. This is a helpful comparison inasmuch as it endorses the range of studies that have highlighted the importance of IDRs as harboring phosphosites.

We thank the reviewer for pointing out that we should include a concise summary of the novel insights that our study revealed. We agree, and to address this, we added such a summary statement in the final paragraph of the discussion.

Page 21-22, lines 30-7: “*By assessing predicted protein structures for almost the entire human proteome, we here integrated PTM information at an unprecedented and comprehensive scale. Gratifyingly, for phosphorylation – the most frequently studied PTM type - our results confirm many previous findings about their structural preferences. They also reveal novel insights that expand to other modification types, exemplified by the observed accumulation of ubiquitination sites in structured regions. With the tools developed here, we have shown that comprehensive, in silico structural information can be used to prioritize physiological PTM sites from in vitro screens. Furthermore, they allowed us to select potentially regulatory sites by extracting PTMs in short IDRs; from phosphorylated kinase activation loops and extending to sequence regions with PTMs that are currently unexplored but likely to be of functional importance. Finally, based on the predicted structures, StructureMap identifies patterns of PTM proximity, revealing potential regulatory mechanisms that occur in 3D space.”*

Among the interesting findings are observations that ubiquitination is preferentially targeted to sites with low sidechain exposure scores. This has important and interesting implications for the impact of unfolding on ubiquitination. In this context, the G3BP2 result is also very interesting. The sites of ubiquitination are localized (mostly) to the NTF2L dimerization / oligomerization domain. This suggests a linkage between the monomer - dimer / oligomer equilibrium and ubiquitination. Accordingly, in addition to a linkage with folding - unfolding equilibria, it follows that ubiquitination might preferentially target folded domains that form higher order oligomers. It would be very interesting to investigate this issue and put the findings on a quantitative footing.

This is a very interesting observation and suggestion. However, a systematic investigation of binding interfaces would be a new project, clearly beyond the scope of this paper. Based on the reviewer’s comment, we added the observation of the ubiquitinated NTF2-like domain to the results and also highlight the possibility of future studies to further integrate protein interaction information to the revised manuscript as follows:

Page 7, lines 16-18: “*Interestingly, six of those ubiquitinations are localized in the NTF2-like domain, which ranges from amino acids 11-133 and plays an important role in G3BP2 oligomerization and the binding of Deubiquitinating enzyme 10 (USP10) [23].”*

Page 20, lines 1-5: “*Coming back to G3BP2, the role of the NTF2-domain as oligomerization and USP10 binding site suggests that the ubiquitination of G3BP2 might impact the formation of protein assemblies. Recent progress in the prediction of protein complex structures will enable a systematic analysis of such effects in future studies [41,42]*.”

While the smoothed pLDDT scores are indeed useful for identifying IDRs, using the specific AlphaFold structure for the IDR can be problematic. Please clarify if this is indeed the case for the calculation of pPSE. If this is the case, then how does one test the validity of the score, seeing as this will be useful for comparative assessments. The problem here is the reduction of an ensemble to a single, essentially randomly drawn structure for the IDR. Comparisons made to date between the static structures and data from in vitro biophysical characterizations of IDRs show that the static structure of an IDR within AlphaFold predictions bears no resemblance to any of the ensembles that have been characterized using measurements integrated with computations. One, hopefully constructive suggestion would be to quantify the extent to which pPSE scores computed using the current method agree with scores computed using ensembles that have been deposited in the PEDB.

We indeed calculate the pPSE values and estimate according IDR stretches based on the AlphaFold predicted structure. However, one essential component of our pPSE metric is that we take the predicted aligned error (PAE) into account. The PAE is generally very small between two amino acids that are within the same structured domain, but very large between (a) amino acids within a domain and amino acids in an IDR and (b) amino acids in two different domains that are linked by an IDR (also see Figure P1 just below).

Figure P1: Screenshot from <https://alphafold.ebi.ac.uk/entry/Q5VSL9> in the ‘Predicted aligned error tutorial’ section.

For amino acids with very low pLDDT scores and known IDRs, the PAE is commonly very large. This means that the pPSE values for such residues almost exclusively considers neighbors in the primary amino acid sequence and the 3D position is neglectable. Using the PAE information therefore enables us to overcome the major shortcomings of using ‘static’ models. In our benchmark, IDR estimation based on the pPSE metric that uses the ‘static’ structure including PAE values performs even better in comparison to only considering the pLDDT values in our benchmark (Figure 1B and 1C, orange vs. purple).

Our results further confirm that the use of PAEs has a clear beneficial impact on the precision and recall of IDR prediction (Figure 1B and 1C, red vs. violet colors). When we benchmarked our results against IDR predictions based on IUPred2, which is purely sequence based, we could show that our metric determines IDRs more confidently.

A comprehensive comparison of IDR predictions derived from AlphaFold structures with ensembles deposited on the PEDB is unfortunately not practical due to following limitations: First, it is to the best of our knowledge not possible to directly extract position errors similar to the PAE values for each residue in the structure. Although one could calculate a similar metric based on the deposited ensembles, this would be a new research project in our opinion – much beyond the scope of this study. Second, PEDB only contains 242 structural ensembles to date. This means that only a relatively small subset of proteins could be investigated, which makes statistical analyses very difficult.

To address the reviewer’s concern, we edited the main text by adding an explanation about the impact of considering the PAE metric when analyzing protein structures predicted by AlphaFold:

Page 4, lines 1-6: “*We found that it is crucial for all structure-based calculations to take AlphaFold’s confidence and PAE metrics into account for best accuracy, as also pointed out before [17]. This ensures that the predicted structures from AlphaFold are not considered as ‘static’, but as estimates with a certain degree of positional uncertainty that varies across the protein sequence. This is particularly important for estimating metrics for intrinsically disordered proteins and proteins with longer IDRs. Therefore, we developed a prediction-aware metric to evaluate amino acid side chain exposure.*”

Please define STY sites. Does this simply mean sites containing Ser, Thr, and Tyr residues?

Yes. We now included a definition of ‘STY sites’ in the main text.

Page 9, lines 7-9: “… *we first confirmed that phosphorylations are generally enriched in kinase phosphorylation motifs compared to all serines, threonines and tyrosines (STY sites) in the proteome.*”

Also, from a semantic and conceptual standpoint, classifying regions as being unstructured is misleading. They are disordered, which has a very different connotation compared to the term unstructured. Please consider not using the latter term because disordered ≠ unstructured. 

We thank the reviewer for pointing this out. We changed the term to *disordered* throughout the manuscript.

The aspect of the study that is concerning is the extraction of 3D hotspots. This is an intriguing hypothesis, although the argument and approach are concerning. Phosphosites are enriched in IDRs. The authors establish this quite clearly. To then remove the IDRs and propose that the phosphosites occur in 3D clusters places a high emphasis on the structurally resolved domains. In effect, as I understand it (and I could be wrong), the analysis suggests that because a region that is the target of PTMs tethers domains that are spatially proximal, it follows that the sites of PTMs form clusters in 3 dimensions. This does not have to follow, although one can see some physical basis for this hypothesis. It is an intriguing hypothesis that merits close scrutiny, perhaps using simulations or by leveraging calculated ensembles deposited in the PEDB. Similar concerns apply to the calculation of distances between modified sites. What is not clear is if the authors are relying on the
static structures provided by AlphaFold. This is highly problematic if true. 

We thank the reviewer for pointing out that we need to describe the procedure for the 3D proximity and cluster analysis in more detail, including its limitations. This is already in part addressed in our comments above. In brief, it is indeed the case that we use ‘static’ structures from AlphaFold for all calculations throughout the manuscript. However, we do take uncertainties into account by adjusting all structure-based proximity evaluations with the predicted aligned errors of each amino acid pair (see Figure P1). This means that uncertainties in the relative positioning of domains that are separated by IDRs are considered in all calculations. While we agree that ensemble structures deposited in PEDB could offer an alternative approach to evaluate the positioning of IDRs and distant domains, the limited number of 242 available ensembles does not allow a proteome-wide analysis.

To address the reviewer’s concerns, we edited the main text to include more details on the computational analysis and its current limitations:

Page 15, lines 23-26: “*Importantly, our metric considers the predicted positional uncertainty between any two PTM sites as a factor in the analysis. This ensures that uncertainties in the relative positioning of folded domains that are linked by flexible IDRs are considered in the proximity calculation.*”

Page 21, lines 17-23: “*The current 3D proximity analysis builds directly on the structures predicted by AlphaFold. This comes with the limitation that IDRs and domains that are linked by IDRs are often not accurately predicted and their relative positions are less defined. In our analysis strategy, we account for this fact by removing long IDRs from the analysis and by considering the PAE for distance calculations. To further evaluate flexible protein regions in this type of analysis, ensemble structures could provide valuable insights, albeit being only available for a small subset of proteins to date (Lazar et al., 2021)*.”


Reviewer #2 (Jon Agirre): 
This article by Bludau and collaborators present an interesting study of AlphaFold-powered structural context for post-translational modifications in proteins at the proteome level. This work employs a similar methodology to what we are doing in my lab with protein glycosylation (for a sneak peek, see Bagdonas, H., Fogarty, C. A., Fadda, E., & Agirre, J., 2021, The case for post-predictional modifications in the AlphaFold Protein Structure Database. Nature Structural & Molecular Biology, 28(11), 869-870), so on the plus side I fully agree with it, and on the negative side I as a reviewer cannot bring an entirely different point of view to the discussion. I believe the decision to use *just* AlphaFold models is justified, as most models will be of at least 'OK' accuracy, and it simplifies the procedure greatly. I think overall there is a good grasp of the technicalities of AlphaFold here, and the limitations are discussed adequately.

We are delighted to hear that Jon Agirre’s team shares our excitement for integrating AlphaFold predictions with PTM data. We added a citation of their work in our introduction.

Page 2, lines 30-31: “*It has further been proposed that AlphaFold has immense potential for incorporating and analyzing PTMs, for instance glycosylation (Bagdonas et al., 2021).*”

I have got a couple of suggestions:

- The lettering on the figures is way too small in many cases (Figs. 4 & 5 for instance).

We increased the font size of Figures 4A, 4G and 5A-D and hope they are more readable now.

- Perhaps I missed it, but I could not find a section on reproducibility. It's great that the source code is available on GitHub, but perhaps the data used for the graphs could also be made available as a zenodo download? 

We agree that reproducibility is very important for scientific publishing. In addition to the already available source code for AlphaMap and StructureMap, we now created a new GitHub repository (https://github.com/MannLabs/structuremap_analysis) which contains all the data and jupyter notebooks to reproduce all analyses and figures presented in the manuscript.

We also edited the main text to guide readers to the repository:

Page 18, lines 27-30: “*To support scientific transparency and reproducibility, we further provide data and python Jupyter notebooks to reproduce all analyses and figures presented herein (https://github.com/MannLabs/structuremap_analysis)*.”


Reviewer #3 (Julian Langer): 
In their manuscript, Bludau et al. present an elegant approach to incorporate information on a variety of PTMs with the prediction of structural information on proteins harboring modification sites. Using previous knowledge on functionally-relevant modification sites (including but not limited to phosphorylation) in combination with deep-learning assisted structure-prediction by alphaFold2, the authors propose strategies to assist in discovery of PTM sites with functional potential, i.e. PTMs within or in proximity to short disordered regions. 
MS-based methods and the ability to detect PTMs has been constantly improving over the last years. However, bioinformatics tools and approaches to systematically analyze and make sense of the plethora of quantified modification sites has not caught up with comparable pace. Hence, the analysis pipeline presented and made accessible for readers in this manuscript is of broad interest for a general audience in molecular, structural and systems biology and thus suitable for publication in PLOS Biology.

We appreciate the thoroughness of the authors' investigation and have only minor comments and suggestions to improve the readability of the manuscript, and two minor biological questions the authors could comment on.

Minor comments:
* Page 4: We think it would be beneficial if the authors would explain the selection of parameters for pPSE calculation in greater detail: How did they decide on 12 Å and an angle of 70°? How robust are the findings with respect to slight changes of these settings?

We thank Julian Langer for the appreciation or our work and for pointing out that the parameter selection needs to be described in more detail. We added a short explanation and a pointer to the methods, which contains a dedicated section “pPSE parameters for estimating amino acid side chain exposure”:

Page 4, lines 18-20: “*We chose these values after considering the average size of amino acids of approximately 3.5 Å and side chain flexibility around the direction of the beta carbon (please refer to the methods section for further details)*.”

The question of robustness regarding the pPSE parameter selection is not trivial to assess. This is one of the reasons why we decided to perform most of the presented enrichment analyses based on the IDR classification rather than on the binary accessibility classification based on the pPSE with 12 Å and 70°. For the IDR prediction, we performed a parameter screen based on a ground truth benchmark. For side chain exposure, we were only able to make an educated guess of feasible parameters which we then evaluated visually for some example proteins. Depending on the selected parameters, different cutoffs need to be selected for both accessibility classification and for visualization. Without ground truth, a comprehensive parameter screen and robustness test was therefore not practical. Overall different parameters might even be sensible for looking at different PTM types such as small chemical modifications (phosphorylation) versus large, bulky ones (ubiquitination). However, independent of these considerations, we expect meaningful results for parameters ranging from ~ 10-25 Å distance and an angle between ~45-90°. We added an explanatory sentence to the method section:

Pages 24, lines 24-29: “*Due to missing ground truth data, we could not perform a comprehensive parameter screen and robustness test. However, based on considerations of the molecular distances involved and on exemplary visualizations, we expect meaningful exposure metrics for parameters ranging from ~ 10-25 Å distance and an angle between ~45-90°. Interpretability then depends on different pPSE thresholds. In future studies, it may be useful to adjust parameters depending on the research question and possibly the type and size of modification types that are being assessed*.”

* Typo: "proteome wide" in title, "proteome-wide" in text

We now consistently adapted the spelling to “proteome-wide” in both title and text.

* Figure 2: It would be helpful, if the authors provided some more details in the axis-labels, i.e. 'p_reg' = regulated phospho-events.

We adjusted axis labels from ‘p_reg’ to ‘p regulatory’ for all modification types. Additionally, we added PhosphoSitePlus and the study by Hansen et al. as the sources of the data.

* Page 6: There seems to be a typo '… ubiquitination sites from (written: form) proteasome-inhibitor-treated …'

We corrected this mistake.

* Page 9: The authors expand on sequence motif analysis by testing the effect of 3D-exposure of the phosphorylation site, they further exploit the Stukalov et al. data for this, but did not mentioned it in the very same paragraph (but before and figure legend). We believe it can be helpful to repeat the source of the data in the first sentence of this paragraph as it prevents the reader to get lost in the text.

We thank the reviewer for pointing this out. The data for the motif analysis actually comes from the study by Sugiyama et al. We highlighted this again in both the Figure legend and the main text.

The revised main text:

Page 9, lines 29-32: “*To further test the effect of 3D exposure filtering on sequence motif analysis in context of the dataset from Sugiyama et al. [2], we selected kinases and performed a motif analysis separately for sites of high surface exposure (pPSE ≤ 5) and sites of low exposure (pPSE > 5) using the PSSMSearch tool [3,4]*.”

The revised caption for Figure 3D:

*“****(D)*** *Sequence logos for different kinases in context of the dataset from Sugiyama et al. [2]. Motifs for phosphosites of high exposure (pPSE ≤ 5) are shown on the left and phosphosites of low exposure (pPSE > 5) are shown on the right. The PSSMSearch tool [3] was used with a log odds scoring method [4]*.”

* Page 10: It would improve readability if the authors added some more description in the figure legend for the motif, i.e. mentioning the selection with respect to surface expose (left) / (right).

We added the pPSE thresholds for high versus low exposure both in the Figure itself and in the legend. Please find the revised caption text in our response to your comment just above.

* Page 13, Figure 4G: Labeling of bar graph on the left (x-axis) could be more detailed with respect to "p_functional_0" and "p_functional_5".

We updated the axis labels from “p” to “p (PhosphoSitePlus)”, from “p_reg” to “p regulatory (PhosphoSitePlus)”, from “p_functional_0” to “p (Ochoa et al.)” and from “p_functional_5” to “p functional ≥ 0.5 (Ochoa et al.)”.

* Page 11+12: Functional score of phospho-sites. Is this derived from Ochoa et al.. It is not clearly mentioned where the authors extracted that piece of information

We added a clarifying statement in the text to ensure that readers understand that the functional scores are derived from Ochoa et al.:

Pages 12-13, lines 30-1: “*Strikingly, 55 of the 79 kinases (70 %) with an overlap of the extended short IDR and the annotated activation loop have an annotated regulatory phosphosite in PhosphoSitePlus or a functional score higher than 0.5 (as determined by Beltrao and co-workers [5]), for a total of 99 different phosphosites*.”

Minor questions:
* Page 6: Given their similarity, could the authors comment on the observed discrepancy between sumoylation and ubiquitination in Figure 2A, esp. for sites lacking functional annotation?

Despite their similarities in sequence and structure, ubiquitinations and sumoylations have very different functions in the cell. One of the main functions of ubiquitination is to tag (misfolded) proteins for degradation whereas sumoylation does not have this role. Therefore, it is not unexpected to observe a discrepancy between the enrichment of modifications in IDRs for ubiquitination and sumoylation.

* Page 7: The analysis of ubiquitination in structured vs. unstructured region is very interesting. It would be interesting to investigate proteomics datasets of linkage-specific ubiquitin-enrichments (K48 vs. K63) to look for potential differences in these two modification with respect to the site of modification.

This is a great suggestion and we have included a statement in the discussion and outlook section to highlight future possibilities to evaluate linkage-specific ubiquitination sites.

Page 20, lines 5-7: “*Further investigations could combine the above analyses with information from linkage-specific ubiquitination studies (for instance, K48 vs. K63) or even information about different ubiquitin side chain architectures to elucidate possible 3D topologies and associated functionalities*.”

The current limitation in proteomics is that linkage specific ubiquitin enrichment protocols are commonly based on enriching entire modified proteins rather than modified peptides. This results in much lower numbers of identified linkage-specific sites compared to the more frequently used diGly enrichment protocol. The limited number of reported sites causes challenges to the statistical evaluation of the results. However, future improvements in the coverage of linkage-specific ubiquitination studies will likely enable us to resolve linkage-specific information.
